# Geometry Optimisation of a Hall-Effect-Based Soft Fingertip for Estimating Orientation of Thin Rectangular Objects

**DOI:** 10.3390/s19184056

**Published:** 2019-09-19

**Authors:** Muhammad Hisyam Rosle, Zhongkui Wang, Shinichi Hirai

**Affiliations:** 1 Department of Robotics, Graduate School of Science and Engineering, Ritsumeikan University, Shiga 525-8577, Japan; 2 Research Organization of Science and Technology, Ritsumeikan University, Shiga 525-8577, Japan

**Keywords:** force sensing, soft robotics, tactile sensor, optimisation

## Abstract

Soft tactile sensors have been applied to robotic grippers for assembly. It is a challenging task to obtain contact information and object orientation using tactile sensors during grasping. Currently, the design of Hall-effect-based tactile sensors to perform such tasks is based on trial and error. We present a method of investigating the optimal geometrical design of a cylindrical soft sensor to increase its sensitivity. The finite element model of a soft fingertip was constructed in Abaqus with two design variables, i.e., hollow radius and magnet position. Then, the model was imported into Isight, with the maximisation of magnet displacement as the objective function. We found that the optimal design was at the boundary of the parameter design space. Four fingertips were fabricated with one intuitive, one optimal, and two optional sets of parameters. Experiments were performed, and object orientation was estimated by utilising linear approximation and a machine learning approach. Good agreements were achieved between optimisation and experiments. The results revealed that the estimated average error in object orientation was decreased by the optimised fingertip design. Furthermore, the 3-axis forces could successfully be estimated based on sensor outputs.

## 1. Introduction

Tactile sensation plays an important role in providing crucial information about the features of contacted objects for robotic systems that perform assembly tasks in uncertain environments. In this rapidly advancing field, the integration of soft tactile sensors with robotic grippers provides a robot system with the advantage of interacting with and perceiving a handled object precisely for successfully performing assembly tasks. Tactile sensors provide useful information, such as applied forces, shapes, and material properties, through physical contact with objects. Research has been conducted on tactile sensors in recent years, such as a flexible tactile sensor that uses a magnetorheological elastomer [1], a tactile sensor for a wire insertion task [2], soft inductive tactile sensors based on the Eddy-current effect [3], and 6-axis force/torque tactile sensors that utilise optoelectronics devices [4]. Moreover, a distributed robotic tactile sensor capable of estimating 3-axis applied forces has been introduced in [5]. These sensors accurately estimate contact force. However, designing tactile sensors that can stably grasp and estimate the orientation of thin rectangular objects, such as handling thin circuit boards in the electrical and electronics industry, is a challenging task. Robotic gripper needs to grasp at the edge of thin circuit boards since the sensitive and small electronics parts are commonly placed on the board surface. The orientation of the objects may vary during the grasping and assembling process due to fingertip deformation and collision between parts. Thus, estimation of the orientation of the grasped objects is crucial to accomplish successful assembly.

The design of a soft tactile fingertip has been proposed in our previous work to address the above issues [6]. We presented a simple-structured soft tactile sensor embedded with a 3-axis Hall sensor and five magnets. A soft gripper was constructed by integrating two such sensors in a parallel configuration, and grasping tests were performed on thin objects. The orientation of the thin objects was described by a nonlinear relationship from sensor outputs. However, this sensor design is intuitive, and sensor performance may vary depending on the geometrical design and the position of embedded magnets in the soft body. Therefore, a geometrical optimisation method is proposed in this paper. Several fingertip designs with various shapes and magnet arrangements were tested, and one suitable design was selected for simulation and fabrication. Then, the selected cylindrical shape of fingertip design was optimised by maximising magnet displacement under contact during grasping. The two design parameters were hollow cavity size and magnet position. Results were validated through comparison with other designs in pushing and grasping tests. In addition, a calibration approach was developed to map sensor outputs to 3-axis contact forces, and the validation test results are presented in this paper. Moreover, the hysteresis and sensitivity of the fingertip are provided. 

The rest of this paper is organised as follows: Section 2 provides a review of related tactile sensors and optimisation techniques. The fingertip design configuration is given in Section 3. The optimisation method of the determined fingertip design is described in Section 4. The experimental procedures are presented in Section 5. Results and discussions on sensor performance are provided in Section 6. Finally, the conclusions and the direction of future work are presented in Section 7. 

## 2. Related Works

Studies on magnetic-based tactile sensors have been actively performed in recent years. This review focuses on magnetic-based tactile sensors that estimate contact forces and object properties, such as shape and orientation. Then, the optimisation methods employed to improve the performance of various tactile sensors are discussed. 

In recent years, studies have investigated the ability of magnetic-based tactile sensors to estimate contact forces. Among these sensors, a pyramid-shaped tactile sensor embedded with a 3-axis Hall element was proposed to estimate normal and shear forces with high accuracy [7]. A mathematical model of 3-axis force approximation based on a cylindrical-shaped tactile sensor was presented in [8]. The application of Hall-effect sensors was further investigated in the development of a distributed tactile sensor for measuring 3-axis forces [9]. A flexible tactile sensor that used a magnetorheological elastomer was introduced in [10] to address the issue of fractures and the poor maintainability of soft coverings. Solid magnets were replaced with a magnetic powder blended with silicone rubber to ensure force estimation without quick saturation and reduce the thickness of a sensing element [11]. In addition, a Hall-effect sensor was applied as a force sensor in surgical applications, such as minimally invasive surgery [12]. In other applications, magnetic-based tactile sensors were utilised for texture and slip detection [13,14,15]. Applications in humanoid robots were reported in [5,16]. Moreover, such tactile sensors were actively applied to detect deformable objects [17] and to produce vectors that represented various objects [9]. Object weight estimation by a robot finger embedded with a Hall-effect sensor was presented in [18]. 

Several optimisation techniques have been proposed to improve the accuracy and performance of tactile sensors. A mathematical objective function that corresponded to sensor accuracy was applied to design an optimal multi-axis force sensor [19]. Then, in the design of a cantilever-based tactile sensor with a piezoresistive stress sensing element, force sensitivity was optimised by introducing a nonconducting island in a p-well [20]. In [21], finite element (FE) analysis was applied to a tactile sensor based on optoelectronic technology to select the optimal sensor geometry sensitive to contact forces. Furthermore, a shape optimisation method that utilised an FE model was proposed to design an optimal mechanically decoupled 6-axis force sensor consisting of 24 strain gauges [22]. In the micromanipulation field, a soft tactile sensor array was optimised by FE analysis using COMSOL Multiphysics software, which optimised the geometry and placement of liquid microchannels in the elastic sensor body to estimate submillimetre contact localisation and forces [23]. An optimisation method based on Abaqus and Isight was presented to study the optimal design of soft pneumatic actuators in [24,25]. The application of such tools provides a useful solution for identifying the design with the best parameters among potential candidates. However, despite the mentioned research, the geometrical optimisation of Hall-effect-based tactile sensors has not been investigated yet. Thus, in this study, we investigated the optimal design of a Hall-effect-based soft tactile sensor. To decrease the footprint of the fingertip, installing magnetic-based tactile sensors are more preferable due to its simple structure and small IC package, so it is more feasible for the gripper compared to strain gauges [22] and other vision-based tactile sensors [26]. Moreover, the fabrication of the fingertip is much easier, i.e., by 3D printing all parts except magnets and IC package, compared to other tactile sensors that require much complex fabrication process [21,23]. In addition, the low fabrication cost of the fingertip which was below $40 makes it desirable. In this study, two geometrical parameters of a tactile fingertip were studied, and the objective was to maximise the deformation of embedded magnets. Abaqus and Isight were employed to construct an optimisation framework. Then, the optimised fingertip was used to estimate the orientation of thin objects, particularly, a thin rectangular circuit board. 

The main contributions of this work are as follows: (1) a method of optimising the geometry of Hall-effect-based tactile sensors by maximising embedded magnet displacement is developed using Abaqus and ISight; (2) a calibration method is built to estimate 3-axis forces based on magnetic flux density (MFD) changes; (3) experimental validation is conducted to evaluate the optimised tactile fingertip. 

## 3. Configuration Design of Soft Fingertip

**Figure 1:**
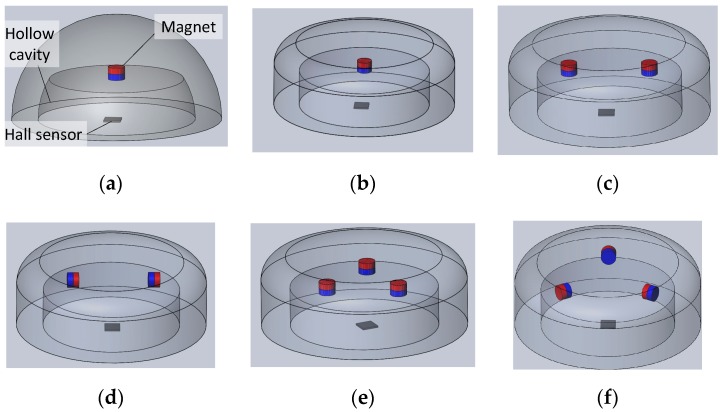

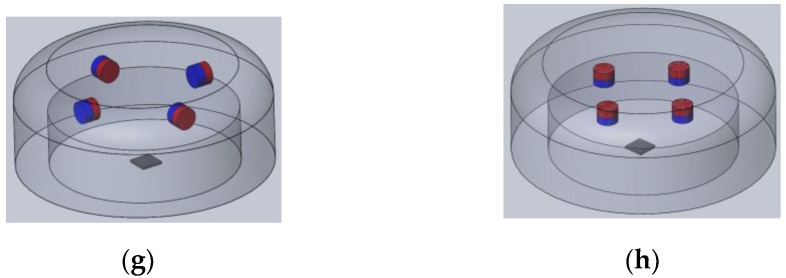
Several structures of soft fingertips consisting of one 3-axis Hall sensor and magnet(s): (**a**) Design I; (**b**) Design II; (**c**) Design III; (**d**) Design IV; (**e**) Design V; (**f**) Design VI; (**g**) Design VII; and (**h**) Design VIII.

We constructed eight different designs (Design I–VIII) to determine the suitable shape of a fingertip that could sense object orientation, as shown in Figure 1. The structural designs of the fingertip were different in terms of fingertip shape (hemispherical and cylindrical shape) and the arrangement of the magnets embedded inside the soft body. The higher number of magnets (more than four) can cause the magnets to repel or attract each other due to smaller distances between them. Thus, the number of magnets was limited to four. 

**Figure 2:**
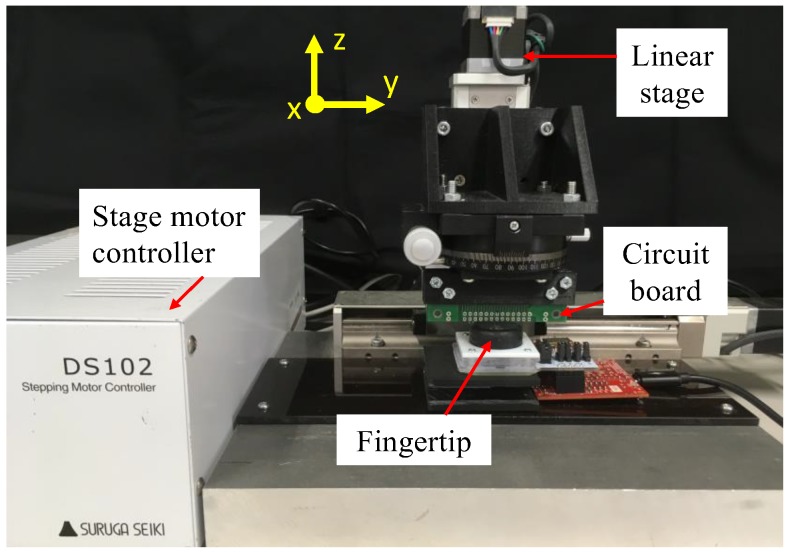
Experimental setup for pushing test with various soft fingertip designs.

All fingertip designs were investigated using pushing tests. The test setup is shown in Figure 2. The fingertip was connected to a platform board (AKDP Rev.D7, Asahi Kasei, Tokyo), and the MFD value of a 3-axis Hall sensor was taken as the initial value. Then, a circuit board fixed to a linear stage was pushed on top of the soft fingertip with different orientations around the z-axis. The initial value was subtracted from the recorded MFD value. Based on the MFD recorded for different orientations, we determined the suitable design for the soft fingertip to be optimised.

**Figure 3:**
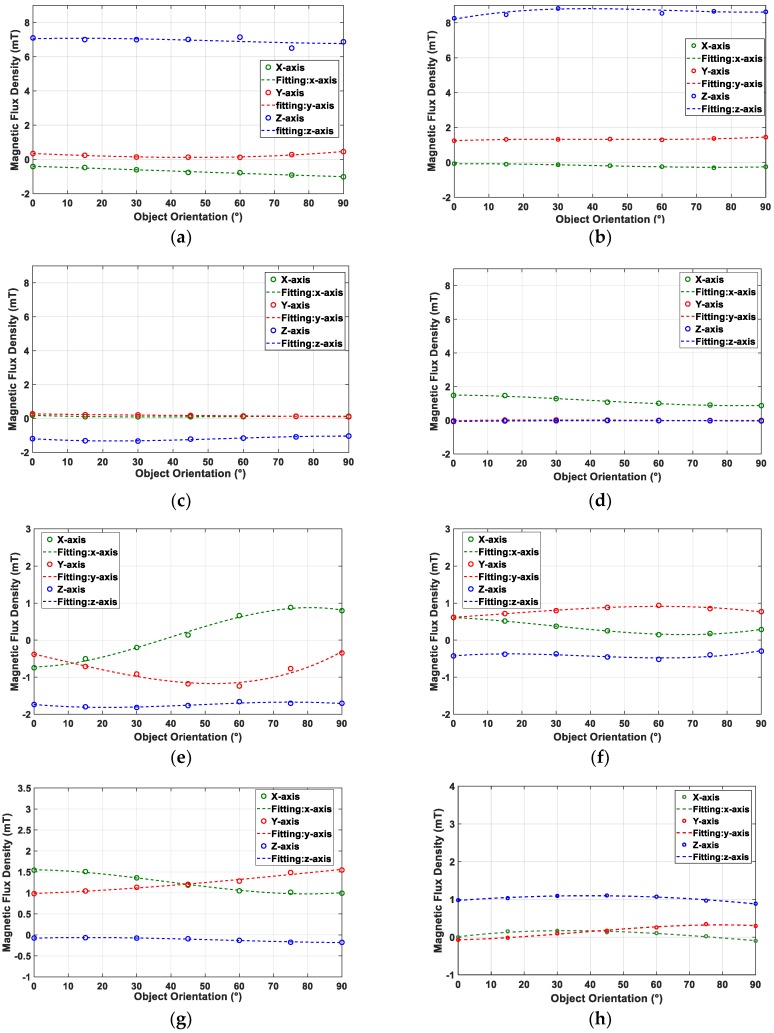
Changes in magnetic flux density (MFD) at different object orientations for each fingertip design: (**a**) Design I; (**b**) Design II; (**c**) Design III; (**d**) Design IV; (**e**) Design V; (**f**) Design VI; (**g**) Design VII; and (**h**) Design VIII.

Figure 3 shows the MFD values obtained for different object orientations for each fingertip design. Figure 3a–d,h show that the MFD value did not change significantly with object orientation. Therefore, we concluded that Design I, II, III, IV, and VIII could not distinguish the orientation of the object that pushed on the fingertip. Next, Figure 3e–g depict that the MFD value changed significantly with object orientation. Therefore, we concluded that Design V–VII could estimate object orientation. We selected Design VII because the higher number of magnets embedded inside the fingertip can provide a larger distribution of magnets inside the soft body. As the sensitivity of the tactile sensor depends on magnet deformation, the larger distribution of magnets in the soft body generates a more sensitive response from the fingertip. 

**Figure 4:**
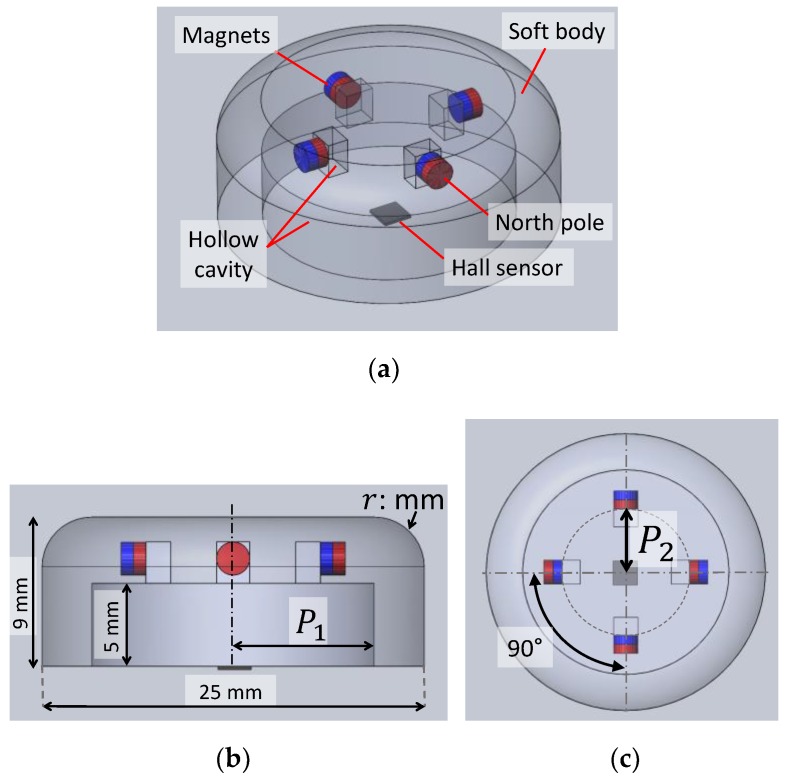
Intuitive fingertip design: (**a**) isometric view, (**b**) side view with parameter *P*_1_ indicating hollow radius, and (**c**) top view with parameter *P*_2_ indicating the magnet position.

The proposed soft fingertip is shown in Figure 4. One 3-axis Hall sensor was fixed at the sensor base, and four cylindrical neodymium magnets with a size of Ø2 mm × 1.5 mm were embedded in the soft body. To stably manipulate the circuit board, we considered that sensor diameter should be at least 0.2 times the length (*l*) of the circuit board and sensor height should be at least 0.1 times the width (*w*) of the board. The expected circuit board size is 125 mm in length and 90 mm in width. Therefore, we determined that the tactile sensor was 25 mm in diameter (*d*) and 9 mm in height (*h*), as shown in Figure 4b. We parameterised the design of the soft tactile sensor using variable *P*_1_ to represent the hollow radius (Figure 4b). To simplify the optimisation problem, the height of the hollow cavity and the fillet size at the edge of the sensor (*r*) were defined to be constant at 5 mm and 3 mm, respectively. Additionally, the distance of the magnets from the centre of the sensor was parameterised as *P*_2_ (Figure 4c). Therefore, we employed parameters *P*_1_ and *P*_2_ to optimise the design of the sensor. The intuitive values of these parameters were set as *P*_1_ = 9 mm and *P*_2_ = 5.5 mm.

## 4. Design Optimisation and Fingertip Fabrication

### 4.1. Finite Element Simulation

**Figure 5:**
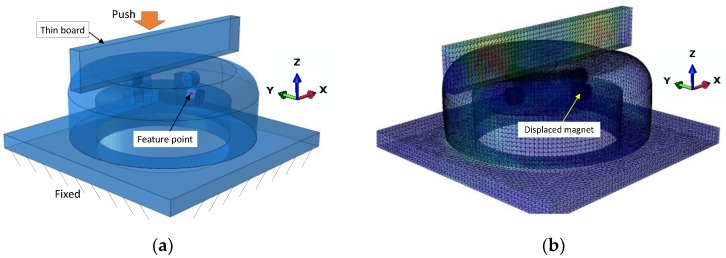
Simulation result: (**a**) initial condition and (**b**) fingertip deformation under a push displacement of 1.0 mm of thin board onto the fingertip.

After determining the shape and arrangement of the Hall sensor and magnets, the soft fingertip model was constructed in Abaqus (Dassault System, Waltham, MA, USA) for simulation. In the previous work [6], from the calibration test, we found that the displacement of the magnet along z-axis shows approximately linear relationship to the MFD. Therefore, the maximisation of the magnet displacement was set as the objective function. The FE model of deformation was structured to simulate the displacement of the fingertip geometry under pushing by a thin rectangular object. The soft body and hard material (thin board, magnets, and hard base) were set as linear elastic materials. Young’s modulus was set to be 0.59 MPa and 2000 MPa to simulate the material properties of the TangoBlackPlus and VeroWhite materials of a 3D printer, respectively. The Poisson’s ratio of the soft body was set as 0.48 because of its incompressible characteristic. The density of the soft body was set as 1120 kg/m^3^ by referring the datasheet of TangoBlackPlus material. All parts were meshed using 4-node tetrahedron elements. The interaction between the soft body and board was defined as tangential and normal behaviour by utilising a penalty method with a friction coefficient of 0.7. The connection between the magnets and soft body was defined as a tie constraint. A boundary condition was applied at the base surface to fix the position of the sensor base. The implicit dynamic simulation of the pushing motion of the board onto the soft fingertip with a displacement of 1 mm was implemented. We defined a feature point at the centre of the magnet surface (Figure 5a). The deformation of the feature point was recorded for further optimisation. The simulation result is shown in Figure 5b. Based on the result, it was found that the feature point was displaced by 0.56 mm along the z-axis. 

### 4.2. Optimisation Framework

**Figure 6:**
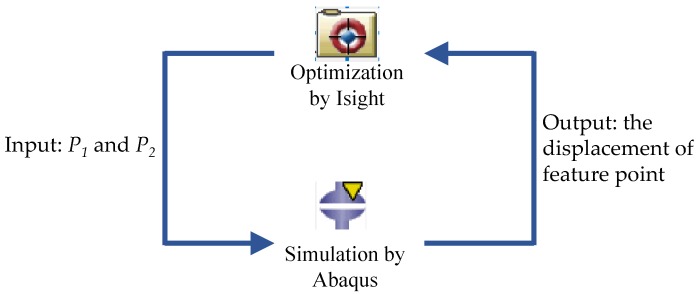
Optimisation process of the fingertip using Isight and Abaqus software.

The optimisation was performed using Isight (Dassault System, Waltham, MA, USA). The FE model constructed in Abaqus was combined within an optimization Isight workflow, as shown in Figure 6. The aim of the optimisation was to maximise the displacement of the magnet within the range of the predetermined parameter sets. The two variables (*P*_1_ and *P*_2_) defined in the Abaqus model were imported as input parameters. The objective function was set as the maximisation the displacement of the feature point along the z-axis. In Isight, we employed ‘pointer’ as the optimisation technique. This technique controls a set of standard algorithms to solve optimisation problems. The range of parameter *P*_1_ was set as [8.0, 10.0] with an increment of 0.1, and the range of parameter *P*_2_ was set as [4.5, 6.5] with an increment of 0.5. The minimum value of parameter *P*_2_ was set to 4.5 mm because a value smaller than 4.0 mm will cause the magnets to attract or repel each other. Isight iterated the FE simulation, updated the parameters (*P*_1_ and *P*_2_), and, finally, returned the optimal parameters.

### 4.3. Optimisation Results

**Figure 7:**
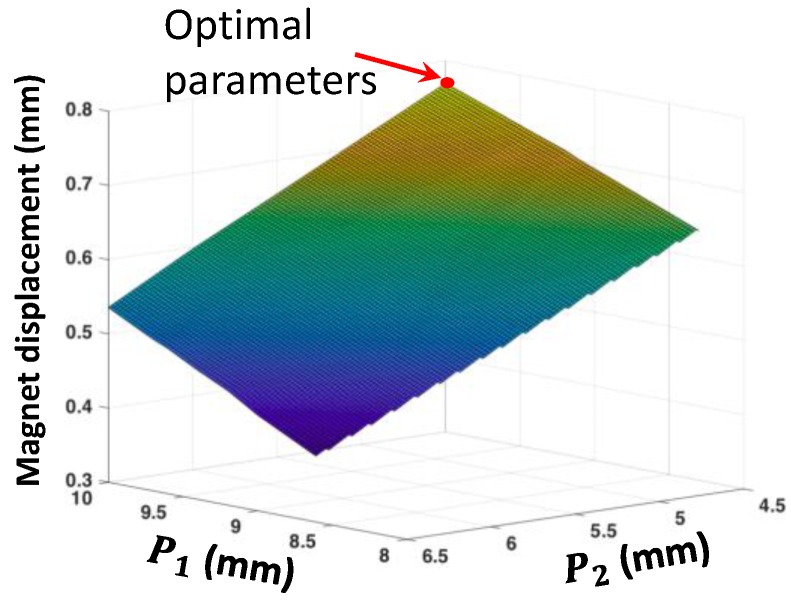
Optimisation result: a surface plot of magnet displacement versus hollow radius *P*_1_ and magnet position *P*_2_. The optimal parameters were found at the boundaries of the parameter design space.

In the optimisation process, Isight iterated the FE simulation 27 times. The optimal design was found at parameter values of *P*_1_ = 10 mm and *P*_2_ = 4.5 mm. The objective function (feature point displacement) started from approximately 0.562 mm and ended at the largest deformation of 0.770 mm. The objective function was plotted against the input parameters (*P*_1_ and *P*_2_), as shown in Figure 7. We found that the optimal design of the fingertip was at the boundaries of the parameter design space. Table 1 shows the simulation results of the objective function with different parameter values. The first set of parameters signifies the intuitive design. The second and third sets indicate intermediate designs. The last set of parameters shows the optimal design of the fingertip.

### 4.4. Fingertip Fabrication

**Figure 8:**
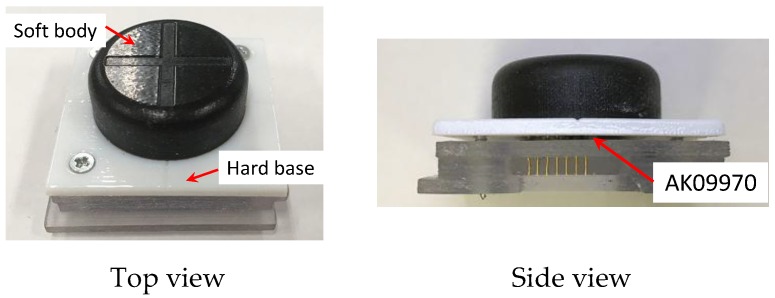
Fabricated fingertip.

The fabricated fingertip is shown in Figure 8. To maintain the performance and repeatability of the sensor, the soft body of the fingertip was 3D printed using the TangoBlackPlus material with a Shore hardness of A27. The hard base of the fingertip was fabricated using the VeroWhite material. The soft body and hard base were fabricated simultaneously by a 3D printer (Objet350, Stratasys, Minneapolis, MN, USA). According to the proposed arrangement illustrated in Figure 4a, we embedded four neodymium magnets (2 mm in diameter and 1.5 mm in height) inside the soft body with a strong adhesive. Four design candidates of the fingertip, i.e., Design *A*–*D*, were fabricated with the different parameters listed in Table 1. 

## 5. Experiments

### 5.1. Pushing Test with Different Object Orientations

#### 5.1.1. Experimental Process

The pushing tests evaluated the performance of the fingertip in estimating the orientation of the object pushed on the fingertip. The same experimental setup (Figure 2) as that described above was implemented. All four designs of fingertips were experimentally examined using the pushing tests. The circuit board was rotated every 15° around the z-axis. For each orientation, the board was pushed to a depth of 3 mm along the z-axis with a velocity of 1 mm/s. The MFD values were recorded at a measurement frequency of 20 Hz. Ten trials were conducted to generate a calibration data set, and five trials were conducted to create the validation data set. 

#### 5.1.2. Calibration Method

We utilised two calibration methods to estimate object orientation based on the output of the Hall-effect sensor, i.e., the polynomial fitting method (PFM) and a neural network, and compared the results obtained using both methods.

A. Polynomial Fitting Method (PFM)

The outputs of the Hall-effect sensor along the x, y, and z axes are given by *S_x_*, *S_y_*, and *S_z_*, respectively. Several experimental data sets are established, where $S_{x_{n}}$–$S_{z_{n}}$ are the outputs of the sensor for *n*-time trials with different object orientations. The relationship between the changes in MFD measured by the Hall-effect sensor and object orientation *φ* is defined by a polynomial function.

(1) $$\varphi =S_{t}A_{t},$$

where *S_t_* and *A_t_* are defined as

(2) $$S_{t}=\left[\begin{array}{cccccccccc} S_{x}^{3} & S_{x}^{2} & S_{x} & S_{y}^{3} & S_{y}^{2} & S_{y} & S_{z}^{3} & S_{z}^{2} & S_{z} & 1\\ \vdots & \vdots & \vdots & \vdots & \vdots & \vdots & \vdots & \vdots & \vdots & \vdots \\ S_{x_{n}}^{3} & S_{x_{n}}^{2} & S_{x_{n}} & S_{y_{n}}^{3} & S_{y_{n}}^{2} & S_{y_{n}} & S_{z_{n}}^{3} & S_{z_{n}}^{2} & S_{z_{n}} & 1 \end{array}\right],$$

(3) $$A_{t}={\left[\begin{array}{ccc} \begin{array}{cccc} a_{0} & a_{1} & a_{2} & a_{3} \end{array} & a_{4} & \begin{array}{cccc} a_{5} & a_{6} & a_{7} & \begin{array}{cc} a_{8} & a_{9} \end{array} \end{array} \end{array}\right]}^{T},$$

and, *a*_0_–*a*_9_ denote the coefficients of the polynomial function. The parameters were calculated using MATLAB. We selected a third-order polynomial function for the calibration process because it provides acceptable accuracy of estimation, as well as lower computational complexity (time consumption) compared to the higher-order of polynomial function. The pseudo-inverse method was applied to calculate the coefficients, as given below.

(4) $$A_{t}={\left(S_{t}^{T}S_{t}\right)}^{-1}S^{T}\varphi .$$

The selected polynomial function was calibrated based on 10 trials for each orientation. Then, the data sets of five validation trials were used to evaluate the coefficients of the function.

B. Feedforward Neural Network (FNN)

The relationship between the Hall-effect sensor output and the orientation of the pushed board was described using the machine learning approach of a feedforward neural network (FNN). We applied an FNN with one hidden layer, which contained 10 neurons for performing the calibration process. The network consisted of three inputs and one output. The inputs were the changes in MFD measured by the sensor, and the target outputs were board orientation. The network consisted of 40 weights (30 input weights and 10 layer weights) and 11 biases. We employed the MATLAB Neural Network Toolbox to calculate the parameters. A linear transfer function was utilised in the output layer to estimate the output. The network was trained using the Levenberg–Marquardt backpropagation algorithm with a maximum of 1000 iteration steps. 

### 5.2. Three-Axis Force Estimation and Hysteresis

#### 5.2.1. Force Calibration Setup

**Figure 9:**
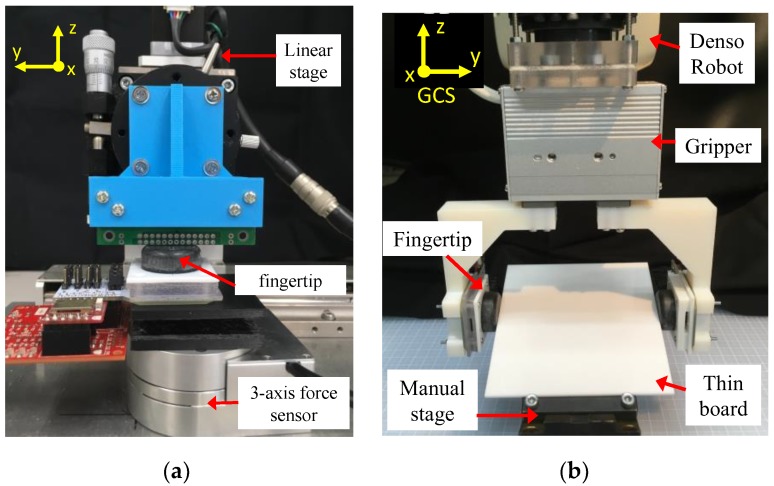
(**a**) Experimental setup for calibration process and (**b**) setup for grasping test.

The calibration process was conducted to estimate 3-axis force. The experimental setup is shown in Figure 9a. The fingertip design with the optimal parameters (*P*_1_ = 10 mm and *P*_2_ = 4.5 mm), i.e., Design *D*, was placed on a stage. Then, the stage was fixed to a 3-axis force sensor (PFS055YA251U6, Leptrino Co. Ltd., Japan) as a reference sensor. Three steps were conducted for the calibration process. In the first step, the thin circuit board was pushed by the linear stage on top of the fingertip in the normal direction (z-axis) to a displacement of 3 mm. The normal force and sensor output were measured during the pushing motion at a frequency of 20 Hz. In the second step, the board was pushed with a normal displacement of 2 mm and a displacement of 2 mm in the direction tangential to the top surface of the fingertip along the x-axis. The shear force along the x-axis and the sensor output were recorded. In the third step, the board was pushed by 2 mm in the normal direction and 2 mm in the direction tangential to the y-axis. The movement speed was set to be 1 mm/s for the first step and 0.5 mm/s for the second and third steps. For all tests, the initial value was subtracted from the measured value to remove offsets. Ten trials were conducted to create the calibration data sets, and five trials were performed to generate the validation data sets. The trained FNN with one hidden layer of 10 neurons was employed to estimate 3-axis force. 

#### 5.2.2. Hysteresis Tests

Hysteresis tests consisting of load and unload displacements in the normal direction (z-axis) were performed to investigate the hysteresis of the fingertip fabricated from the TangoBlackPlus material. An experimental setup similar to that shown in Figure 9a was used. In these tests, the board was pushed onto the fingertip with a speed of 0.25 mm/s for a displacement of 3 mm. After a rest of 1 s, the board was released to the original position with the same speed. The sensor outputs along all three axes were recorded for plotting. Ten trials were carried out, and the average values of sensor outputs were considered. 

### 5.3. Grasping Tests

In the grasping tests, the proposed fingertip was evaluated to estimate the orientation of the grasped object. To investigate the performance of the proposed optimisation method, the tests were conducted using the intuitive design, i.e., Design *A*, and the optimised design, i.e., Design *D*. The test setup is illustrated in Figure 9b. Two fingertips in Design *A* were mounted on the left and right fingers of a robotic gripper (LEHF10K2-32-R16N, SMC Co. Ltd., Japan). The gripper was attached to a 6-axis Denso Robot. A thin acrylic board (100 mm × 100 mm × 2 mm) was applied as the grasped object and placed on a manual rotary stage. The robot was moved to the contact position at the centre of the fingertip surface. We assumed that each grasping task was carried out with the contact region passing through the fingertip surface at the determined contact position. To perform the tests, the board was gripped with a grasping velocity of 5 mm/s and a grasping displacement of 4 mm was applied to it. The changes in MFD were recorded by the sensors for both fingers at a frequency of 20 Hz. The object orientation around the y-axis of the global coordinate system was adjusted manually using the rotary stage. 

Two tests were conducted for evaluating sensor design under the conditions of (1) trained object orientation and (2) untrained object orientation. In the first test, the grasping task was carried out with object orientations of 0°, 10°, 20°, and 30°. Ten trials were conducted at each orientation. Seven data sets were considered for calibration while the remaining three data sets were used for validation. In the second test, the grasping task was performed with untrained object orientations of 5°, 15°, and 25°. Three trials were carried out at each orientation. For both tests, an FNN with one hidden layer consisting of 20 neurons was employed to estimate thin board orientation. For calibration, the changes in the MFD of both sensors (six signals) were considered as inputs and the corresponding object orientations (one output) were set as target outputs. Finally, both tests were repeated using the optimised design.

## 6. Results and Discussion

### 6.1. Pushing Tests with Different Object Orientations

**Figure 10:**
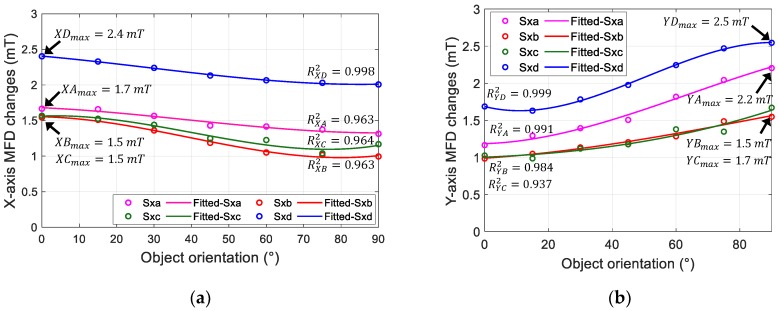
Changes in MFD of sensor output along (**a**) x-axis and (**b**) y-axis when the thin board was pushed on top of the surface with different orientations.

In Figure 10, the changes in MFD measured along the three axes are plotted at each object orientation. A third-order polynomial was used to approximate the MFD value at each object orientation. Then, R-squared values were calculated along the x-axis ($R_{XA}^{2}-R_{XD}^{2}$) and y-axis ($R_{YA}^{2}-R_{YD}^{2}$); these values are shown in the graph. Moreover, the maximum changes in MFD along the x-axis (*XA*_max_ − *XD*_max_) and y-axis (*YA*_max_ − *YD*_max_) that were recorded during pushing motion with different orientations are shown in the graph. It can be observed that Design *D* had the highest MFD value compared to other candidates under a pushing displacement of 3 mm. Furthermore, the R-squared values along the x and y axes demonstrated the best performance for Design *D* compared to other candidates. 

**Figure 11:**
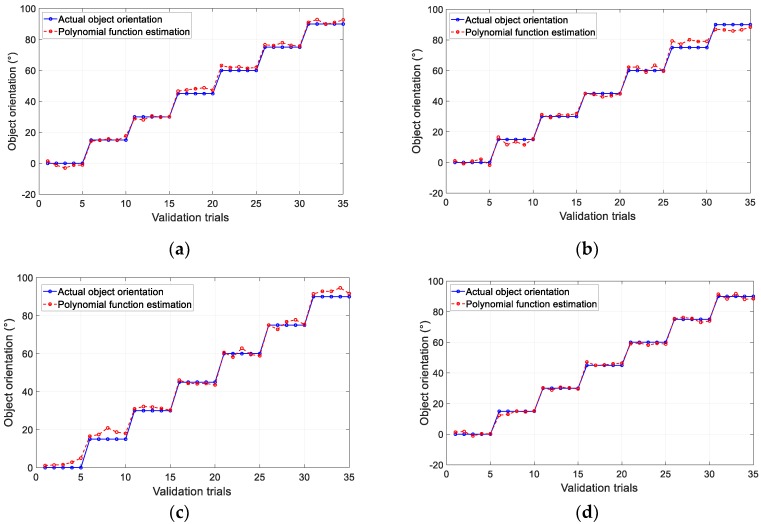
Results of five validation trials for each object orientation using PFM for each design: (**a**) Design A, (**b**) Design B, (**c**) Design C, and (**d**) Design D.

**Figure 12:**
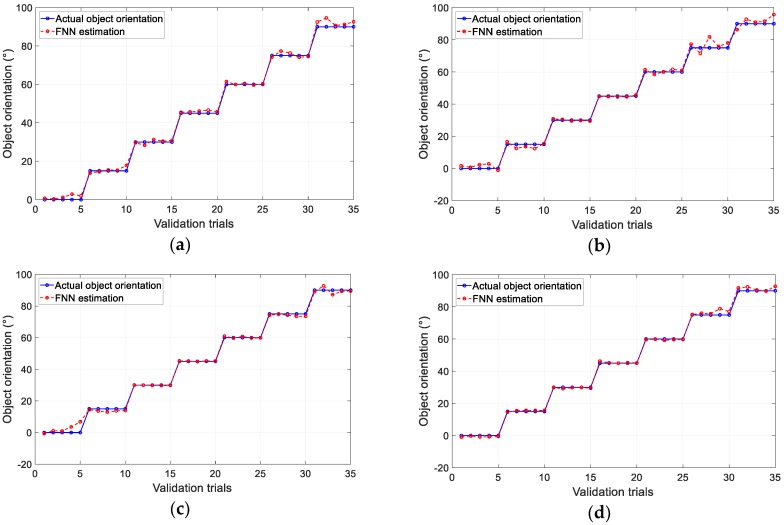
Results of five validation trials for each object orientation using FNN for each design: (**a**) Design A, (**b**) Design B, (**c**) Design C, and (**d**) Design D.

The results of the pushing test performed using the polynomial approximation method for different designs are shown in Figure 11a–d. The results of object orientation estimation by the FNN method are shown in Figure 12 for each design. The graphs show the estimated value of five validation trials for each object orientation at every 15° for both calibration methods. The total average error in the validation trials (35 trials) is provided in Table 2. Based on the results, in both estimation methods, Design *D* had the least error in averaged object orientation compared with other candidates, while Design *B* generated the highest average error. This tendency agreed with the results given in Table 1. This suggested that the fingertip design with the highest magnet displacement (objective function) resulted in the optimal geometry of the tactile fingertip. This demonstrated that an increase in magnet displacement generated more features in the sensor output and therefore resulted in higher sensitivity of the sensor. Overall, the machine learning approach of the FNN method provided the least error in object orientation estimation. Moreover, the average time consumption required for estimation was 82 ms for FNN, that is lower compared to 108 ms for third-order PFM. Thus, we selected the FNN method for the calibration of the proposed soft tactile fingertip.

### 6.2. Three-Axis Force Estimation and Hysteresis

**Figure 13:**
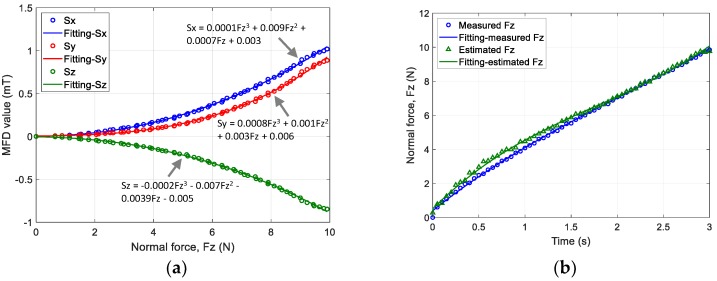
(**a**) MFD value of 3-axis sensor output vs. normal force Fz. (**b**) Measured normal force and normal force estimated using FNN with one hidden layer.

**Figure 14:**
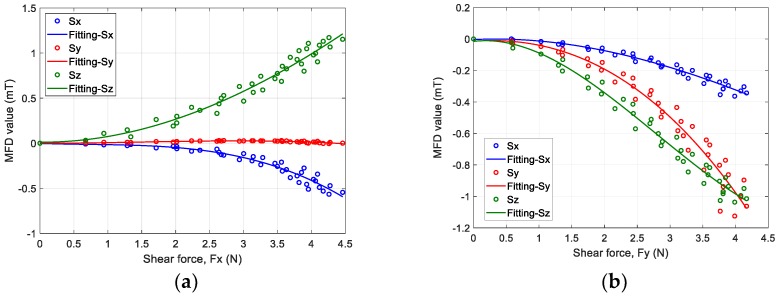
MFD value of 3-axis sensor output vs. shear force along the (**a**) x-axis and (**b**) y-axis.

**Figure 15:**
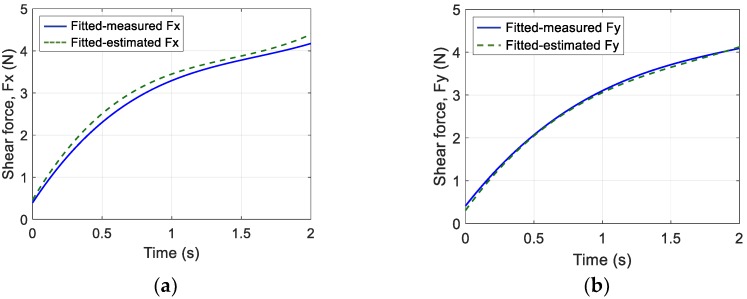
Measured and estimated shear force along the (**a**) x-axis and (**b**) y-axis.

The results obtained from the first step of the z-axis force estimation for Design *D* are shown in Figure 13. The relationship between the change in MFD along each axis and normal force was fitted using a third-order polynomial, as shown in Figure 13a. The normal force estimated using a machine learning approach and the normal force measured until a pushing displacement of 3 mm were compared, as shown in Figure 13b. The graph shows the averaged values of five validation trials. The maximum error between both data sets was 0.71 N. The result proved that the proposed fingertip design based on the suggested estimation method could estimate normal force. Next, the relationship between the changes in the MFD of the sensor output along the x and y axes and shear force is shown in Figure 14a,b, respectively. The sensor outputs along the x axis was decreased, and the outputs along z axis was increased as shear force *F_x_* increased. In addition, the sensor outputs along and y and z axes decreased as shear force *F_y_* increased. The comparison of the estimated and measured shear force along the x and y axes is shown in Figure 15. The estimated and measured shear forces exhibited similar behaviour with time. These results demonstrated good agreement between the proposed calibration method and experimental data. The range of estimated contact forces and sensor performance may depend on the stiffness of the soft body. Previous work [8] reported that increasing the stiffness of the material could increase the maximum normal force. It proves that materials with lower stiffness could positively affect the sensitivity of the sensor; however, slippage could occur due to lower grasping force.

**Figure 16:**
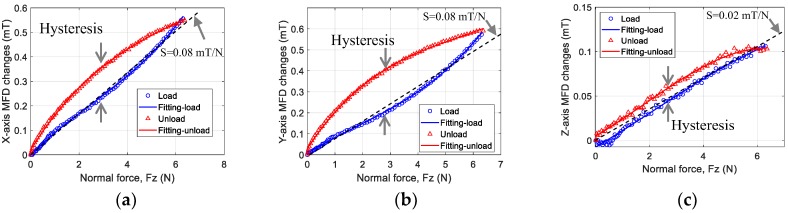
Sensor response curves in hysteresis tests for normal force along the (**a**) x-axis, (**b**) y-axis, and (**c**) z-axis.

The hysteresis of the sensor is shown in Figure 16. The graphs present the measured sensor output against normal force. Hysteresis occurs owing to the soft material characteristic that leads to a nonlinear relationship between each axis of sensor outputs to the normal force during a load and unload task. The sensitivity, *S* (mT/N), of the sensor was calculated during the ascending period indicated by the dashed line in Figure 16 along each axis. The sensitivity was estimated as *S* = 0.08 mT/N along the x and y axes and 0.02 mT/N along the z-axis. 

### 6.3. Grasping Tests

Table 3 shows the results of estimated object orientation in the grasping tests. The average error for the three trials performed at each orientation was calculated. Based on the results, the average errors under the conditions of trained and untrained object orientation for Design *A* were 5° below our target range. In comparison, Design *D* exhibited the least overall average error in both conditions. By taking the overall average error, the estimated error was reduced by approximately 27%, from 2.46° to 1.79°. This proved that the proposed optimisation technique could provide a good solution for optimising tactile fingertip performance. Moreover, it was validated that even with untrained orientation, the network could estimate object orientation within 3° of estimation error for Design *D*. Furthermore, even with a higher number of calibration inputs (six input signals), we found that the estimated error in the grasping tests was slightly larger compared to the pushing tests. In detail, for Design *D*, the estimated average error obtained by the FNN was 0.78° in the pushing tests and 1.78° in the grasping tests. This may be due to the slight displacement of the thin board during the grasping motion, which caused the orientation of the board to differ from the initially desired orientation. As the current limitation, the edge of thin objects has to be in contact with the centre of the fingertip surface during grasping. As the solution, the robotic arm could be adjusted to grasp the objects in the centre to perform the estimation. 

## 7. Conclusions

This paper presented a geometrical optimisation method for a cylindrical soft tactile fingertip embedded with a Hall effect sensor and four neodymium magnets to manipulate thin rectangular objects, e.g., circuit boards. In particular, the optimal values of sensor hollow radius and magnet position were studied. These parameters were assumed to have a strong effect on magnet displacement once the overall size of the sensor and the magnet arrangement were determined. The FE model of the soft fingertip was constructed in Abaqus and was then integrated with an optimisation workflow in Isight. To optimise sensor performance, the maximisation of magnet displacement along the z-axis was set as the objective function in the workflow. The magnet size and arrangement and the overall size of the sensor were maintained constant. The optimisation results suggested that a larger hollow radius and a smaller distance from the magnet to the surface centre generates a larger displacement of the magnet. The intuitive design, optimal design, and other optional designs were fabricated for tests. In the pushing tests, the optimal design (Design *D*) provided the least average error for the PFM and FNN estimation methods. Results showed that the optimised fingertip design demonstrated better performance compared to other designs. Then, a calibration method was proposed to map the changes in the MFD of the sensor output to the applied 3-axis force, and evaluation tests were conducted. The estimation method could be applied in sensing grasping force, where the force is monitored during thin objects’ grasping task. Finally, grasping tests were conducted with intuitive and optimal designs, and object orientation was estimated using the FNN. The grasping test results showed that the optimal design generated a smaller average error compared to the intuitive design, with an estimation error below 3°. The results are promising for the application in robotics systems. As the current geometrical optimisation method was purposely for cylindrical fingertips, new design variables are required if a different shaped sensor is to be applied.

In future work, the optimisation of the overall dimensions of the sensor and magnet will be investigated. In addition, other machine learning methods will be considered to improve the calibration performance of the tactile fingertip. Finally, the effect of magnetic interference will be considered to overcome the crosstalk effect produced by magnetic interference.

## Figures and Tables

**Table 1:** Simulated results of feature point displacement with different parameter values.

No.	Design	*P*_1_ (mm)	*P*_2_ (mm)	Displacement (mm)
1	A	9.0	5.5	0.562
2	B	10.0	6.5	0.536
3	C	8.5	4.5	0.549
4	D	10.0	4.5	0.770

**Table 2:** Average estimated error in total validation trials for each design using polynomial fitting method (PFM) and feedforward neural network (FNN).

Design	Average Error Using PFM (°)	Average Error Using FNN (°)
A	1.584	1.185
B	2.063	1.671
C	1.908	1.044
D	1.052	0.779

**Table 3:** Object orientation estimation results of each design.

		Trained Object Orientation				Untrained Object Orientation		
		0°	10°	20°	30°	5°	15°	25°
Intuitive design, Design *A*	Average error (°)	1.35	1.05	4.15	3.99	4.13	0.51	2.04
	Overall average error (°)	2.64				2.23		
Optimal design, Design *D*	Average error (°)	1.28	2.65	1.42	1.75	1.02	2.45	1.97
	Overall average error (°)	1.78				1.81		
